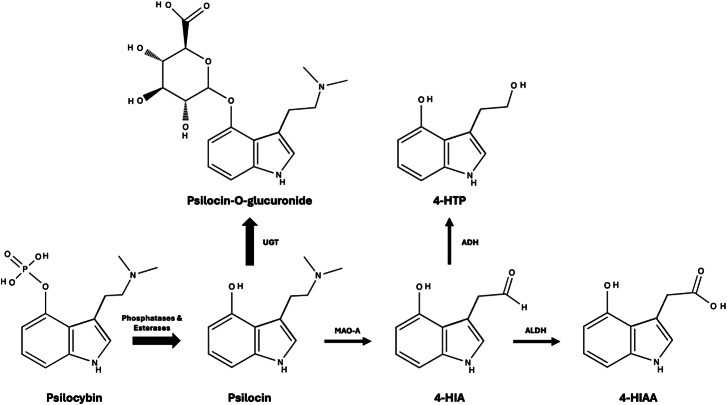# Psilocybin and hallucinogenic mushrooms – ERRATUM

**DOI:** 10.1017/S1092852925100291

**Published:** 2025-06-09

**Authors:** Mathieu Fradet, Carlton M. Kelly, Anna J. Donnelly, Trisha Suppes

Cambridge University Press apologises for an error in the originally published article. In the original version of Figure 3, a nitrogen atom has been incorrectly put in place of a carbon atom. The corrected Figure 3 appears below: